# Genome-Centric Analysis of Microbial Populations Enriched by Hydraulic Fracture Fluid Additives in a Coal Bed Methane Production Well

**DOI:** 10.3389/fmicb.2016.00731

**Published:** 2016-06-08

**Authors:** Steven J. Robbins, Paul N. Evans, Donovan H. Parks, Suzanne D. Golding, Gene W. Tyson

**Affiliations:** ^1^Australian Centre for Ecogenomics, School of Chemistry and Molecular Biosciences, The University of QueenslandBrisbane, QLD, Australia; ^2^School of Earth Sciences, The University of QueenslandBrisbane, QLD, Australia

**Keywords:** coal bed methane, aminicenantes, OP8, phycisphaerae, methane, hydraulic fracturing, metagenomics

## Abstract

Coal bed methane (CBM) is generated primarily through the microbial degradation of coal. Despite a limited understanding of the microorganisms responsible for this process, there is significant interest in developing methods to stimulate additional methane production from CBM wells. Physical techniques including hydraulic fracture stimulation are commonly applied to CBM wells, however the effects of specific additives contained in hydraulic fracture fluids on native CBM microbial communities are poorly understood. Here, metagenomic sequencing was applied to the formation waters of a hydraulically fractured and several non-fractured CBM production wells to determine the effect of this stimulation technique on the *in-situ* microbial community. The hydraulically fractured well was dominated by two microbial populations belonging to the class Phycisphaerae (within phylum Planctomycetes) and candidate phylum Aminicenantes. Populations from these phyla were absent or present at extremely low abundance in non-fractured CBM wells. Detailed metabolic reconstruction of near-complete genomes from these populations showed that their high relative abundance in the hydraulically fractured CBM well could be explained by the introduction of additional carbon sources, electron acceptors, and biocides contained in the hydraulic fracture fluid.

## Introduction

Over the last decade, coal bed methane (CBM) has emerged as an important resource for meeting rising global energy demands. It is anticipated that consumption of natural gas will grow by 1.5% each year until 2040, the fastest growth of any fossil fuel resource (U.S. Energy Information Administration, 2013). CBM is generated through biotic and abiotic processes, however analysis of methane isotopic compositions from CBM reservoirs worldwide suggest that the majority of methane is derived from microbial activity, especially at shallow depths (Scott, 2002; Strąpoć et al., 2011; Golding et al., 2013). Despite its economic importance, our understanding of the microbial communities responsible for the conversion of coal to methane is limited, hampering our ability to engineer strategies for stimulating native microbial communities to produce additional methane.

To extract CBM, a vertical well is drilled 200–1000 m into a coal bed. Water and gas are simultaneously extracted from the well and the gas is separated from the water at the surface. In cases where the natural permeability of the coal does not allow for economical rates of extraction, stimulation techniques such as hydraulic fracture are commonly applied. Hydraulic fracture involves the injection of a fluid mixture into the well at high pressure to fracture the coal (Australian Department of the Environment, 2014). The hydraulic fracture fluid mix often contains biocides to inhibit the growth of undesirable microorganisms, namely sulfate reducers, which may cause corrosion of the well bore. Flow paths created by the new fractures are held open by a proppant (e.g., sand, ceramic, or walnut husks) contained in the fracturing fluid. A gelling agent, typically a polysaccharide polymer, is commonly included in the hydraulic fracture fluid to suspend the proppant to ensure that it disperses evenly within the seam. In order to remove the fracturing fluid from the well, a breaker (e.g., hydrogen peroxide, diammonium peroxydisulfate, or a hemicellulase enzyme) is added to the well to depolymerize the gelling agent. Once the fracturing fluid is removed from the well, a production pump is installed at the wellhead to begin dewatering of the CSG well.

Here, community profiles for 11 wells from across the Surat Basin were subjected to metagenomic sequencing and characterization to identify strategies to enhance CBM production. One well, PK-28, had been subjected to hydrofracture stimulation and showed clear differences in community composition to the other wells sampled. The use of additives such as gelling agents, breakers, and biocides in the hydraulic fracture process is commonplace, but it is unknown how these additives may affect CBM community structure. Sugar polymer-based gelling agents and sulfate-based breakers may enable the growth of microorganisms capable of using these compounds, while additives such as biocides are likely to select for specific microbial populations. Metabolic reconstruction of microbial populations enriched in the PK-28 well strongly suggest that this shift in community composition is the result of exposure to hydrofracture fluid additives.

## Materials and methods

### Sample collection

Biomass was collected from 11 previously characterized CBM production wells in the Surat Basin, Australia for metagenomic characterization (Evans et al., 2015). Water chemistry and isotope measurements were also collected for comparison and are described in detail by Baublys et al. (2015). Prior to microbial sampling, temperature, pH, and conductivity were measured using an Accumet multimeter (Fisher Scientific model 13636AP85). When these readings stabilized (~10–20 min), between 10 and 50 l of production water were filtered through two sequential 142 mm stainless steel filter housings (#YY3014236, Millipore, MA, USA) containing a 20 μm polypropylene prefilter followed by a 0.22 μm nitrocellulose filter. Both filters were folded aseptically, placed into separate falcon tubes, and frozen on dry ice for transport back to the laboratory.

### DNA extraction, sequencing, and binning

Metagenomic libraries were prepared using the Illumina Nextera XT DNA Sample Preparation kit and sequenced on two-fifths of a lane on the Illumina HiSeq2000 platform in rapid mode (2 × 100 bp paired end; 500 bp fragment size) producing an average of 4.1 Gb of paired-end data for each sample. Adapter clipping and merging of overlapping reads was performed using SeqPrep v2013-12-17 (https://github.com/jstjohn/SeqPrep). Nesoni v0.99 (https://github.com/VictorianBioinformaticsConsortium/nesoni) was used to remove homopolymers, quality trim bases with a Phred score <20, and discarding trimmed reads ≤30 bp. Assembly of the metagenome was performed using CLC Genomics Workbench v6.5 using default parameters.

Microbial community profiles for all metagenomes were generated by identifying sequencing reads from the 16S rRNA gene and mapping them to the Greengenes database using CommunityM (https://github.com/dparks1134/CommunityM) at a 97% threshold to define OTUs. Binning of the PK-28 metagenome was carried out using DBB v1.0.0 (https://github.com/dparks1134/DBB), which recruits scaffolds into population genomes based on similarity in GC-content, coverage, and tetranucleotide frequency. Genome completeness and contamination were estimated using the CheckM v0.9.6 lineage-specific workflow using default parameters (Parks et al., 2015).

### Statistical analysis

A heatmap showing the relative abundances of all OTUs present at a minimum of 1% in at least one sample was generated using STAMP (Parks et al., 2014). All statistical analyses were performed in R v3.1.2 (R Core Team, 2013). Differences in OTU composition were further explored through principal components analysis of Hellinger transformed OTU relative abundances (Legendre and Gallagher, 2001) using the CRAN package vegan (Dixon, 2003).

### Phylogenetic identification of population genomes

In order to determine the phylogenetic affiliation of each metagenome bin, an approximate maximum-likelihood phylogenetic tree was constructed using FastTree v2.1.7 (Price et al., 2010) from a concatenated set of 83 bacterial single-copy marker genes (Soo et al., 2014) extracted from all PK-28 population genomes ≥70% completeness with ≤10% contamination as well as all IMG v4.0 genomes (Markowitz et al., 2012). Single-copy marker genes were identified and extracted from genomes using HMMER v3.1 (Finn et al., 2011). True maximum-likelihood trees were then re-inferred with RAxML (Stamatakis, 2006) including only IMG genomes of interest from 100 bootstrap replicates, using the PROTGAMMAWAG substitution model.

Maximum likelihood trees were also constructed with RAxML from 16S rRNA gene sequences recovered from previous studies using the GTRGAMMA substitution model. For one recovered population, *Aminicenantes-*PK28, the 16S rRNA gene tree was constructed for the *Aminicenantes* phylum from 100 bootstrap replicates using near-full length (>1400 bp) 16S rRNA genes recovered in previous studies (Rinke et al., 2013; Farag et al., 2014; Gies et al., 2014; Sharon et al., 2015). The 16S rRNA gene sequences obtained from Sharon et al. (2015) were mined from a metagenome where an *Aminicenantes* population genome was recovered, but the 16S rRNA gene and population genome could not be linked (Sharon et al., 2015). Consequently, all four recovered 16S rRNA gene fragments identified in the metagenome were included in the tree. A 16S rRNA gene fragment (~250 bp) extracted from the *Aminicenentates*-PK28 population genome was placed into the full length reference tree by parsimony insertion in ARB (Ludwig et al., 2004). Members of the phylum *Acidobacteria* were used as an outgroup based on a previous analysis showing this phylum to be a sister group to the *Aminicenantes* (Rinke et al., 2013).

### Population genome annotation and metabolic reconstruction

Population genomes were annotated using Prokka v1.8 (Seemann, 2014) and IMG v4.1 (Markowitz et al., 2012). In parallel, open reading frames (ORFs) were identified using Prodigal v2.60 (Hyatt et al., 2010) and the resulting protein sequences were compared to Uniref90 (Suzek et al., 2007), COG (Tatusov et al., 2003), Pfam (Finn et al., 2014), and TIGRfam (Haft et al., 2013) using BLASTP (Altschul et al., 1997) and HMMER (Finn et al., 2011), respectively. Carbohydrate active enzymes (CAZY) were identified with dbCAN (Yin et al., 2012) using BLASTP (Altschul et al., 1997) with an *e*-value cut-off of 1e^−10^ and a coverage fraction cut-off of 0.5. Peptidase families were identified by searching sequences annotated as peptidases against the MEROPS peptidase database using BLASTP (Rawlings et al., 2014). A sequence was assigned to the protein family of its best hit as long as the hit had an *e*-value threshold of ≤1e^−20^.

## Results

### CBM formation water sampling and community profiling from metagenomes

**Figure 1:**
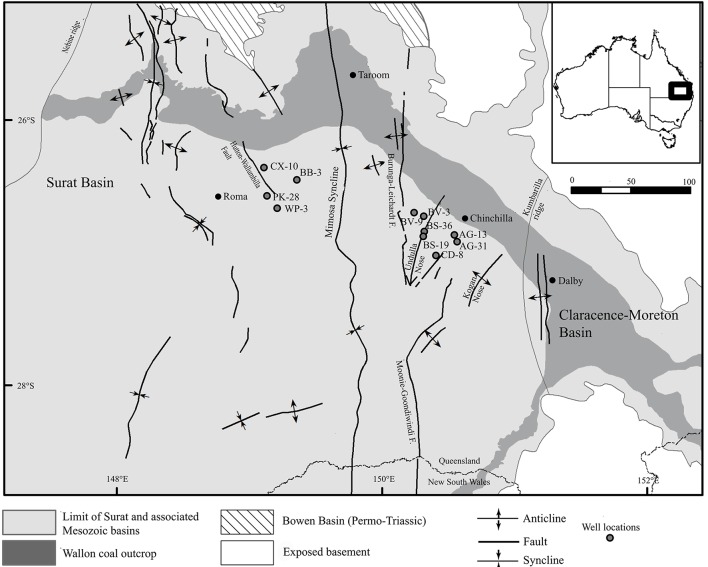
**Map of the Surat Basin (Queensland, Australia) showing the locations of CBM production wells sampled for metagenomic sequencing**. Map modified from Hamilton et al. (2014).

**Figure 2:**
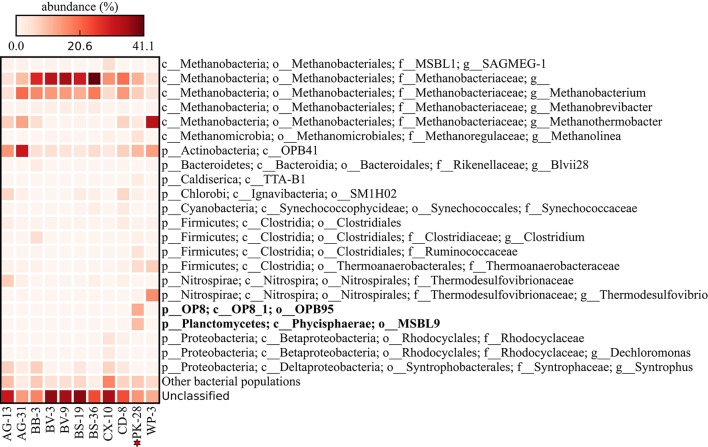
**Heatmap of the relative abundance of community members (operational taxonomic unit; OTU) from each of eleven formation waters sampled for microbial community profiling**. Each row represents an OTU clustered at 97% identity. Only OTUs present at ≥1% relative abundance in at least one sample are shown. Reads that did not match the reference database at ≥97% identity were designated as unmapped.

**Figure 3:**
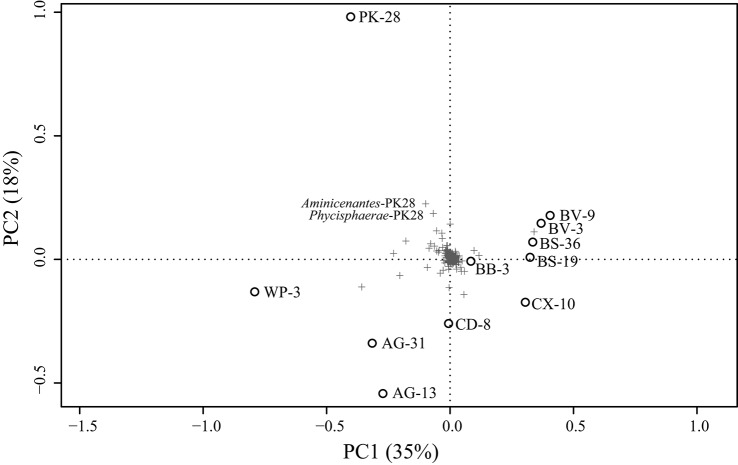
**PCA of Hellinger transformed OTU relative abundances for each formation water sample**. Clustering of PK-28 away from the other wells appears to be driven by the abundance of *Phycisphaerae*-PK28 and *Aminicenantes*-PK28 which are not present at >0.1% in any other well (Figure 2). Plus signs represent individual OTUs and circles represent well samples.

Metagenomic datasets averaging 4.1 ± 0.6 Gb of paired-end data were generated for formation waters collected from 11 CBM wells located in the Surat Basin, Queensland, Australia (Figure 1; Table 1). One of these wells, PK-28, had been subjected to hydraulic fracture stimulation in September of 2011. The hydraulic fracture fluid was injected and removed after ~2 weeks. However, no gas or water was extracted from PK-28 well until July 2013, approximately 4 months prior to sampling. In order to identify differences in the microbial community composition of the hydraulically fractured and non-fractured wells, community profiles for each formation water sample were generated by classifying 16S rRNA gene sequences from the metagenomic datasets (Figure 2). Operational taxonomic units (OTUs) from the actinobacterial order OPB41 (2–30%) and methanogens from the *Euryarchaeotal* family *Methanobacteriaceae* (0–39%) were typically dominant in all wells. In contrast, the PK-28 microbial community was dominated by OTUs belonging to the *Planctomycetes* class *Phycisphaerae* (9%), the candidate phylum *Aminicenantes* order OPB95 (11%), the actinobacterial order OPB41 (10%), and hydrogenotrophic methanogens from the family *Methanobacteriaceae* (11%). Comparison of the PK-28 community composition to that of the other wells using principal components analysis showed that PK-28 clustered away from the other wells, indicating that its overall microbial community was atypical compared to the rest of the basin (Figure 3). The difference in the PK-28 community composition was primarily driven by the *Aminicenantes* and *Phycisphaerae* populations. The Aminicenantes were identified only in wells BB-3, WP-3, and BV-9 while the *Phycisphaerae* were identified in all wells other than WP-3 and BV-9. However, they only reached an abundance of >0.1% in PK-28. Wells WP-3 and AG-13 also appeared to be somewhat atypical compared to the rest of the basin (Figure 3). These wells showed higher abundances of thermophilic populations from the family *Thermodesulfovibrionaceae* and genus *Methanothermobacter*, as well as a higher abundance of the class OPB41.

**Table 1:** **CBM wells sampled from the Surat Basin**.

**Site ID**	**Site name**	**Latitude**	**Longitude**
AG-13	Argyle 13	−26.9134	150.4656
AG-31	Argyle 31	−26.8991	150.4606
BB-3	Ben Bow 3	−26.4411	149.3893
BV-3	Bellevue 3	−26.7275	150.3090
BV-9	Bellevue 9	−26.7104	150.2720
BS-19	Berwyndale South 19	−26.8675	150.3086
BS-36	Berwyndale South 36	−26.8546	150.3103
CD-8	Codie 8	−27.0061	150.3916
CX-10	Coxon Creek 10	−26.3686	149.0963
PK-28	Pickanjinnie 28	−26.5821	149.1209
WP-3	Washpool Creek 3	−26.6407	149.2323

### PK-28 population genome binning

*De novo* assembly of the paired-end data for PK-28 produced 52,312 scaffolds ≥500 bp with an N50 value of 3831 bp. A total of 11 population genomes with ≥70% completeness and ≤10% contamination were obtained by partitioning scaffolds based on GC-content, tetranucleotide frequency, and coverage (Table 2). These genomes span the majority of dominant populations identified in the 16S rRNA gene community profile, with the exception of *Caldiserica*. The coverage of the population genomes generally matched the expected relative abundances, with coverage being highest for members of the family *Methanobacteriaceae*, followed by the *Phycisphaerae* and *Aminicenantes*. The *Aminicenantes* (*Aminicenantes-*PK28), and *Phycisphaerae* (*Phycisphaerae*-PK28) population genomes were targeted for detailed metabolic characterization to determine why these microorganisms were enriched in the hydraulically fractured well. Both the Aminicenantes-PK28 and Phycisphaerae-PK28 population genomes have been deposited in IMG under IDs 2593339135 and 2593339136 respectively.

**Table 2:** **Phylogenetic identification of population genomes and estimates of genome completeness and contamination**.

**Population genome ID**	**Phylum**	**Lowest taxonomic classification**	**% Completeness**	**% Contamination**	**# Scaffolds**	**Genome size (Mbp)**	**% GC**	**Coverage**
0	*Planctomycetes*	c__*Phycisphaerae*	95.5	0	31	2.9	55.3	373
1	*Ignavibacteriae*	p__*Ignavibacteriae*	96.9	2.2	73	3.1	41.6	36
2	*Firmicutes*	f__*Peptococcaceae*	91.1	0.5	36	2.0	62.3	113
3	*Aminicenantes*	p__*Aminicenantes*	88.0	2.5	73	2.5	53.5	301
5	*Actinobacteria*	p__*Actinobacteria*	79.6	1.1	41	1.5	67.8	177
6	*Euryarchaeota*	g__*Methanothermobacter*	92.4	1.4	52	1.6	50.0	31
8	*Euryarchaeota*	f__*Methanobacteriaceae*	100	0	18	1.5	40.6	450
9	*Proteobacteria*	c__*Deltaproteobacteria*	76.7	6.3	170	2.2	64.9	42
10	*Euryarchaeota*	g__*Methanothermobacter*	90.5	1.6	44	1.6	49.2	67
11	*Proteobacteria*	f__*Methanomicrobiaceae*	78.1	0.7	44	1.5	57.5	175
12	*Firmicutes*	o__*Clostridiales*	85.3	8.4	130	1.7	48.6	15

### Phylogenetic placement of phycisphaerae-PK28 population genome

**Figure 4:**
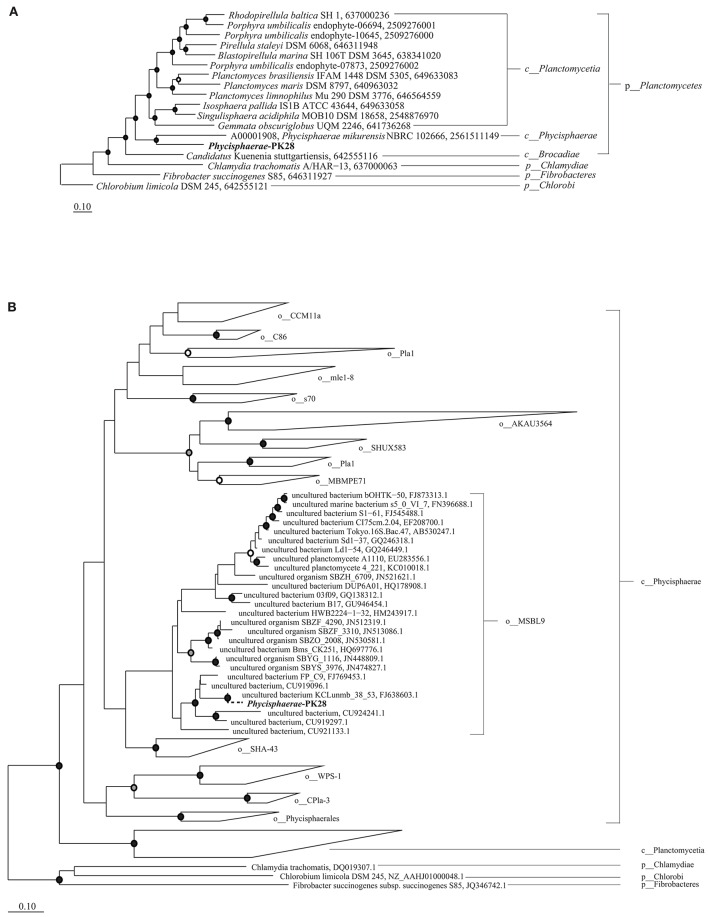
**Maximum likelihood phylogenetic trees showing the placement of *Phycisphaerae*-PK28 within the phylum *Planctomycetes* using (A) a concatenated set of 83 bacterial single-copy marker genes and (B) 16S rRNA gene sequences from *Phycisphaerae-*PK28 and members of the phylum *Planctomycetes***. These analyses place *Phycisphaerae-*PK28 into the order MSBL9 within the class *Phycisphaerae*. NCBI accession numbers and IMG genome IDs are listed to the right of each sequence. In both trees, white, gray, and black circles represent nodes with 70–80%, 80–90%, and >90% bootstrap support values respectively. For the concatenated marker gene tree, members of the phyla Chlamydiae, Fibrobacter, and Chlorobi were used to root the tree.

The approximate maximum-likelihood phylogenetic tree constructed with FastTree placed *Phycisphaerae-*PK28 (Table 2) within the *Planctomycetes* phylum next to *Phycisphaerae mikurensis* (Fukunaga et al., 2009). A true maximum likelihood tree inferred using all IMG *Phycisphaerae* genomes confirms this placement (Figure 4A). In order to more precisely determine its taxonomic affiliation, a 16S rRNA gene tree was constructed (Figure 4B) from the full-length rRNA gene sequence from *Phycisphaerae-*PK28 and additional *Planctomycete* sequences obtained from the Greengenes database (Desantis et al., 2006). This analysis placed *Phycisphaerae-*PK28 in the candidate order MSBL9.

### Phylogenetic placement of aminicenantes-PK28 population genome

**Figure 5:**
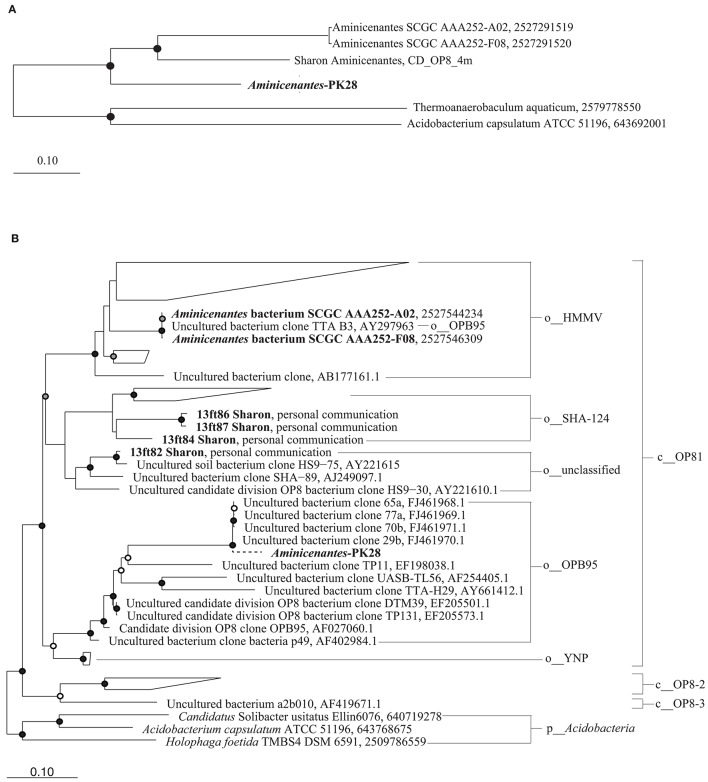
**Maximum likelihood phylogenetic trees constructed from (A) 83 bacterial single-copy marker genes and (B) near-full length 16S rRNA gene sequences obtained from the sequence read archive (Farag et al., 2014), as well as from sequenced genomes from Rifle Creek (Sharon et al., 2015) and Sakinaw Lake (Gies et al., 2014)**. Only 16S rRNA gene sequences >1400 bp were included in order to ensure overlap in alignment with the short fragment from *Aminicenantes*-PK28. A dashed line is used to indicate that this sequence was inserted by maximum parsimony. This analysis places *Aminicenantes*-PK28 into the order OPB95 within the class OP8-1. White, gray, and black circles represent nodes with 70–80%, 80–90%, and >90% bootstrap support values respectively.

The approximate maximum-likelihood phylogenetic tree constructed with FastTree placed *Aminicenantes*-PK28 within the candidate phylum *Aminicenantes* (Figure 5A). Three *Aminicenantes* genomes have been sequenced to date (Rinke et al., 2013; Sharon et al., 2015), but there are no cultured representatives of this lineage. Previous phylogenetic analysis of the *Aminicenantes* using 16S rRNA gene sequences (>800 bp in length) identified several putative subgroups within the candidate phylum, including four proposed classes and eight orders (Farag et al., 2014). Reconstruction of this phylogeny with the addition of 16S rRNA gene sequences from the three publically available *Aminicenantes* genomes revealed that these genomes belong to two distinct orders, HMMV and SHA-124, within the class OP8-1 (Figure 5B). Parsimony insertion of a 16S rRNA gene fragment from the *Aminicenantes-*PK28 population genome places it within the order OPB95, within the proposed class OP8-1.

### Carbon metabolism

**Figure 6:**
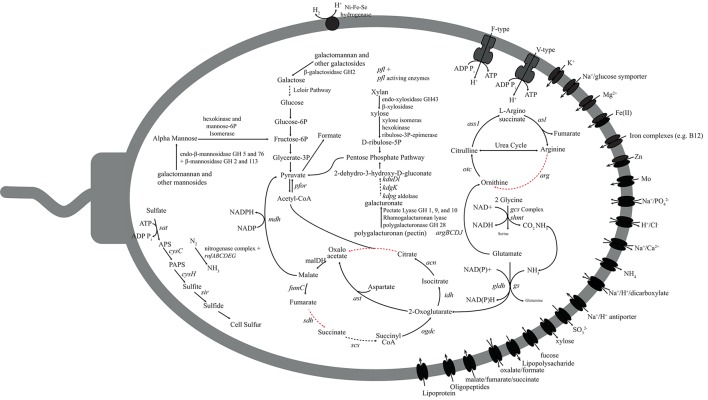
**Metabolic reconstruction of the *Phycisphaerae*-PK28 population genome**. Sugar polymers such as galactomannan (guar), xylan (hemicellulose), and polygalacturonan (pectin) may be degraded and directed toward glycolysis for energy production. The pyruvate generated through glycolysis is likely to be converted to formate by pyruvate-formate lyase. Nitrogen and sulfur could be acquired through nitrogen fixation and assimilatory sulfate reduction. Abbreviations used: *fumC*, fumarate hydratase; *sdh*, succinate dehydrogenase; *scs*, succinyl-CoA synthetase; *ogdc*, oxoglutarate dehydrogenase complex; *idh*, isocitrate dehydrogenase; *can*, citrate hydro-lyase; *cs*, citrate synthase; *ass1*, argininosuccinate synthase; *otc*, ornithine transcarbamylase; *arg*, arginase; *asl*, argininosuccinate lyase; *pfor*, pyruvate-ferrodoxin oxidoreductase; *mdh*, malate dehydrogenase; *pfl*, pyruvate-formate lyase; *rnf*, Rnf electron transport complex; *ast*, aspartate transaminase; *gs*, glutamate synthetase; *gldh*, glutamate dehydrogenase; *gcs*, glycine cleavage system; *shmt*, serine hydroxmethyltransferase; *argB*, acetylglutamate kinase; *argC*, N-acetyl-glutamate semialdehyde dehydrogenase; *argD*, N-acetylornithine aminotransferase; *argJ*, arginine biosynthesis bifunctional protein; *sat*, sulfate adenylyltransferase; *cysC*, adenylylsuflate kinase; *cysH*, phosphoadenylylsufate reductase; and *sir*, assimilatory sulfite reductase.

**Figure 7:**
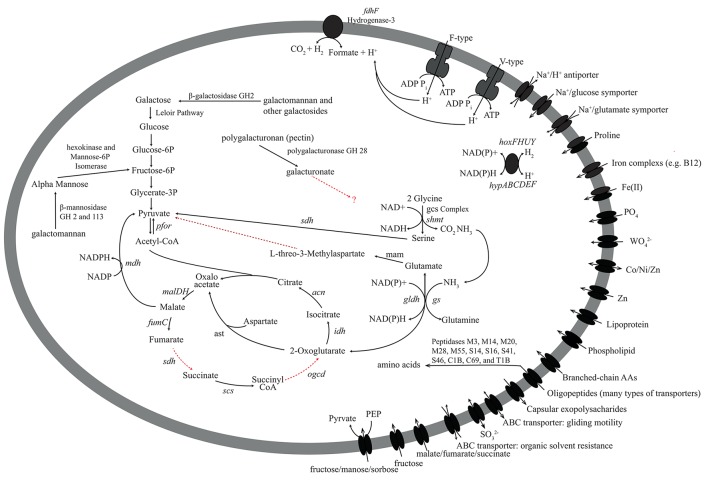
**Metabolic reconstruction of the *Aminicenantes*-PK28 population genome**. The sugar polymers galactomannan (guar) and polygalacturonan (pectin) may be degraded. Galactomannan is likely to be directed toward glycolysis for energy production. Although polygalacturonases were identified, it is not clear how *Aminicenantes*-PK28 processes galacturonate. Several peptidases involved in the degradation of oligopeptides were identified, including family M23, which is involved in the lysis of bacterial cells. These findings suggest that *Aminicenantes*-PK28 may scavenge non-viable cells. Abbreviations used: *fumC*, fumarate hydratase; *sdh*, succinate dehydrogenase; *scs*, succinyl-CoA synthetase; *ogdc*, oxoglutarate dehydrogenase complex; *idh*, isocitrate dehydrogenase; *can*, citrate hydro-lyase; *cs*, citrate synthase; *mdh*, malate dehydrogenase; *pfor*, pyruvate-ferrodoxin oxidoreductase; *ast*, aspartate transaminase; *gs*, glutamate synthetase; *gldh*, glutamate dehydrogenase; *gcs*, glycine cleavage system; *shmt*, serine hydroxmethyltransferase; *sdh*, serine dehydrogenase; *fdh*, formate dehydrogenase.

Differences in the PK-28 well community could result from the introduction of additional carbon sources in the hydraulic fracture fluid, enriching microorganisms best able to utilize the foreign organic matter. The vast majority of fluid is made up of water and inorganic proppant. In addition, the galactomannan polymer guar was used as a gelling agent. In order to determine whether the introduction of the galactomannon contributed to the enrichment of the *Aminicenantes* and *Phycisphaerae* groups, the presence of genes for the utilization of galactomannon as a carbon substrate were examined (Figures 6, 7). All genes required for the endo-hydrolysis of the mannan backbone of galactomannan (endo-βi-mannanase) were identified in *Phycisphaerae*-PK28 (Figure 6), but not *Aminicenantes*-PK28 (Figure 7), and included representatives of glycosyl hydrolase (GH) families 5 and 76. In contrast, genes for the hydrolysis of terminal mannose residues (i.e., β-mannosidase) were identified in both population genomes, including GH families 2 and 113. The presence of β-galactosidases from GH family 2 in both population genomes, and GH 16 in *Phycisphaerae*-PK28, suggests that both *Aminicenantes*-PK28 and *Phycisphaerae*-PK28 are able to cleave the galactose side groups from guar.

Hydrolysed mannose and galactose residues are likely to be fed into glycolysis. For example, hexokinase and mannose-6P-isomerase in both microorganisms can be used to convert mannose to fructose-6P, an intermediate in glycolysis. In *Aminicenantes*-PK28, metabolism of galactose follows the Leloir pathway, whereby β-D-galactose is converted to UDP-glucose by galactose mutarotase, galactokinase, galactose-1-phosphate uridylyltransferase, and UDP-galactose-4-epimerase. Although neither galactose-1-phosphate uridylyltransferase or UDP-galactose-4-epimerase were identified in *Phycisphaerae*-PK28, the presence of galactose mutarotase and galactokinase, as well as a sodium/galactose symporter, suggest that a route similar to the Leloir pathway is used to degrade galactose. In both microorganisms, the pyruvate generated through glycolytic degradation of mannose and galactose may be converted to acetyl-CoA by the action of pyruvate-ferredoxin oxidoreductase for use in a number of biosynthetic reactions. Alternatively, the presence of pyruvate-formate lyase in *Phycisphaerae*-PK28 suggests that pyruvate may instead be converted to formate. Although no specific mechanism for generating formate was found in *Aminicenantes*-PK28, putative genes for formate dehydrogenase (i.e., hydrogenase-3 and formate hydrogenylase) were identified and could be used to convert formate to hydrogen and carbon dioxide as terminal products of fermentation.

### Alternative sugar substrates

In general, both *Aminicenante*s-PK28 and *Phycisphaerae*-PK28 appear to be adapted to utilizing a variety of complex sugar polymers. An analysis of glycosyl hydrolases (GHs), carbohydrate binding modules (CBMs), carbohydrate esterases (CEs), and polysacharaide lyases (PLs) in all PK28 population bins revealed that both *Aminicenantes*-PK-28 and *Phycisphaerae*-PK28 contained higher proportions of carbohydrate active enzymes compared to other members of the PK-28 microbial community, suggesting that they are highly adapted to utilizing sugar polymers as a carbon and energy source (Table 3). *Aminicenantes*-PK28 and *Ignavibacteriae*-PK28 (population genome 1) also devoted a high proportion of their genome to carbohydrates degradation. However, *Ignavibacteriae*-PK28 was not present at high proportion in the PK-28 microbial community (~1.5%).

**Table 3:** **Summary of the total number of carbohydrate-active enzymes in each population genome, including glycosyl hydrolases, carbohydrate esterases, polysaccharide lyases, and enzymes containing carbohydrate binding modules**.

**Population genome ID**	**Lowest taxonomic classification**	**Glycosyl hydrolases**		**Carbohydrate binding modules**		
		**Total GH hits**	**% GH ORFs**	**Total CBM hits**	**% CBM ORFs**	**Total ORFs**
0	c__*Phycisphaerae*	113	4.69	39	1.62	2410
1	p__*Ignavibacteriae*	89	3.15	24	0.85	2822
2	f__*Peptococcaceae*	9	0.47	10	0.52	1908
3	p__*Aminicenantes*	42	2.12	15	0.76	1981
5	p__*Actinobacteria*	4	0.27	3	0.20	1472
6	g__*Methanothermobacter*	1	0.06	1	0.06	1637
8	f__*Methanobacteriaceae*	2	0.13	0	0	1593
9	c__*Deltaproteobacteria*	12	0.61	1	0.05	1972
10	g__*Methanothermobacter*	1	0.06	0	0	1676
11	f__*Methanomicrobiaceae*	0	0	1	0.06	1589
12	o__*Clostridiales*	8	0.45	12	0.67	1796
**Population genome ID**	**Lowest taxonomic classification**	**Carbohydrate esterases**		**Polysaccharide lyases**		
		**Total CE hits**	**% CE ORFs**	**Total PL hits**	**% PL ORFs**	**Total ORFs**
0	c__*Phycisphaerae*	17	0.71	13	0.54	2410
1	p__*Ignavibacteriae*	15	0.53	7	0.25	2822
2	f__*Peptococcaceae*	6	0.31	0	0	1908
3	p__*Aminicenantes*	10	0.50	0	0	1981
5	p__*Actinobacteria*	2	0.14	1	0.07	1472
6	g__*Methanothermobacter*	2	0.12	0	0	1637
8	f__*Methanobacteriaceae*	0	0	0	0	1593
9	c__*Deltaproteobacteria*	2	0.10	0	0	1972
10	g__*Methanothermobacter*	0	0	0	0	1676
11	f__*Methanomicrobiaceae*	0	0	0	0	1589
12	o__*Clostridiales*	4	0.22	0	0	1796

The total number of gene hits to each category is shown, as well as the percentage of all open reading frames (ORFs) devoted to that carbohydrate active enzyme (CAZY) category.

Several genes encoding pectin-degrading enzymes were identified in both *Aminicenantes*-PK28 and *Phycisphaerae*-PK28, although the carbohydrate substrate range of *Phycisphaerae*-PK28 appeared to be more diverse. In both microorganisms, genes encoding polygalacturonases from GH family 28 were identified that could be used to cleave pectin into galacturonate monomers. However, in *Phycisphaerae*-PK28, multiple rhamnogalacturonan and pectate lyases from families 1, 9, and 10 were also identified that could be used to degrade alternative forms of pectin. Although it is unclear how *Aminicenantes*-PK28 processes the galacturonate residues released from pectin cleavage, the presence of genes encoding 5-keto-4-deoxyuronate isomerase (*kduL*), 2-dehydro-3-deoxy-D-gluconate 5-dehydrogenase (*kduD*), 2-keto-3-deoxygluconate kinase (*kdgk*), and 2-keto-3-deoxy-6-phosphogluconate aldolase (*kdpg*) in *Phycisphaerae*-PK28 suggest that galacturoate is likely to be converted to 2-dehydro-3-hydroxy-D-gluconate for use in the pentose phosphate pathway. Additionally, the presence of endo-acting xylanases from GH family 43, as well as β-xylosidases capable of cleaving terminal xylose residues, suggest that *Phycisphaerae*-PK28 is able to hydrolyse xylan. Xylose monomers liberated from this process can be converted by xylose isomerase (*xylA*), hexokinase, and ribulose-3P-epimerase (*rpe*) to D-ribulose-5P, an intermediate in the pentose phosphate pathway that can be directed into glycolysis.

### Amino acid metabolism

Oligopeptide transporters are present in both *Aminicenantes*-PK28 and *Phycisphaerae*-PK28, and both microorganisms appear to be able to utilize select amino acids, such as glycine (glycine cleavage system), glutamate (glutamate dehydrogenase, *gldh*; and glutamine synthetase, *gs*), and aspartate (aspartate transaminase, *ast*). Additionally, genes encoding multiple proline transporters (ABC-type and proline permease) were also identified in *Aminicenantes*-PK28. The presence of genes encoding pyrroline-5-carboxylate reductase (*pcra*) and aspartate transaminase (*ast*) suggest that proline is converted to glyoxylate and pyruvate. In *Phycisphaerae*-PK28, nearly all of the 21 peptidases identified were linked to cell signaling or the modification/maturation of specific proteins, rather than peptide degradation. In contrast, 36 of the 80 peptidases identified in *Aminicenantes*-PK28 are associated with the degradation of oligopeptides, including representatives from peptidase families M3, M14, M20, M28, M55, S14, S16, S41, S46, C1B, C69, and T1B. Interestingly, five genes encoding representatives of peptidase family M23 used to degrade the cell walls of other bacteria were identified in *Aminicenantes*-PK28, which suggests a possible role in peptide scavenging from dead cells.

### Nitrogen, sulfur, and oxygen metabolism

In order to determine whether *Aminicenantes*-PK28 or *Phycisphaerae*-PK28 could carry out either aerobic or anaerobic respiration, the presence of genes for oxidative phosphorylation (electron transport cytochromes), dissimilatory sulfate and sulfite reduction (dissimilatory sulfite reductase; *dsr*), and dissimilatory nitrate and nitrite reduction (dissimilatory nitrate, *nar*; or nitrite reductase, *nrf*) were examined. The absence of these genes suggests that neither *Aminicenantes*-PK28 nor *Phycisphaerae*-PK28 is able to respire using these electron acceptors. However, genes for assimilatory acquisition of sulfur and nitrogen acquisition were identified. For example, genes for assimilatory sulfate reduction were present in both microorganisms, including sulfate adenylyltransferase (*sat*), adenylylsuflate kinase (*cysC*), phosphoadenylylsufate reductase (*cysH*), and a putative assimilatory sulfite reductase (*sir*). Although sulfate may be present in low concentrations in coal strata, peroxidisulfate was also introduced as a breaker to depolymerize the guar gelling agent and may contribute to the cell sulfur pool. The presence of a full operon for an iron-molybdenum nitrogenase (*nif*) was identified, as well as a nitrogenase-associated *rnf* electron transport complex, suggests that *Phycisphaerae*-PK28 is able to fix nitrogen.

### Effect of biocide

Kathon, a mixture of 5-chloro-2-methyl-4-isothiazolin-3-one and 2-methyl-4-isothiazolin-3-one, was included in the fracturing fluid to inhibit microbial growth. The chemical mechanism of this biocide is complex, but is known to act by disrupting the cell membrane, cleave thiol bonds, generate free radicals, and inactivate a number of key metabolic enzymes, including pyruvate dehydrogenase, 2-oxoglutarate dehydrogenase, succinate dehydrogenase, NADH dehydrogenase, lactate dehydrogenase, and alcohol dehydrogenase (Williams, 2007). Of these enzymes, *Aminicenantes*-PK28 and *Phycisphaerae*-PK28 appear to contain only genes for 2-oxoglutarate dehydrogenase.

### Water chemistry and isotopic analysis

Geochemical parameters with the potential to influence microbial community structure were measured (Table 4) as part of a larger investigation into the geochemistry of Surat Basin CBM production waters (Baublys et al., 2015). Some wells were sampled at multiple time points as part of a time series, with one time point paired with samples for microbial analysis. Few systematic trends were evident across the basin, but carbonate tended to be lower in wells located in the western Surat Basin (avg. 1031) compared to the east (avg. 1787). The pH of the wells ranged from 7.6 in well WP-3 to 8.67 in CX-10. Most wells showed temperature values of ~35°C, with wells AG-13, AG-31, WP-3, and PK-28 reaching above 40°C. Conductivity showed more variability, ranging from 4.40 mS in AG-13 to 13.41 mS in WP-3, indicating substantially higher salinity in that well. Consistent with this finding, WP-3 also shows the highest levels of sodium, chloride, potassium, magnesium, and total iron. As described by Baublys et al. (2015), trends within the isotopic data (Table 5) are primarily reflective of the region from which the water is derived. Consistent with the injection of additional water and carbon into the well, PK-28 shows a younger water age than any other well and a higher percentage of modern carbon.

**Table 4:** **Water chemistry CBM production waters**.

**Site ID**	**Date sampled**	**pH**	**Conductance (mS)**	**Temperature (°C)**	**HCO_3_**	**Cl**	**Ca**	**Mg**	**Na**	**K**	**F**	**Total N**	**Total P**	**Total Fe**	**Dissolved Fe**	**Depth (m)**
AG-13*	11/27/2013	8.21	4.40	41.3	2121	443	3	<1	1020	4	4.6	0.9	0.03	0.70	0.08	261–615
AG-31	5/08/2013	8.29	5.5	44.5	1841	799	4	1	1140	5	4.1	0.7	0.04	0.35	<0.05	265–604
AG-31*	11/27/2013	8.24	5.02	32.1	1865	790	4	1	1150	5	3.8	0.9	0.02	0.46	0.18	265–604
BB-3*	11/25/2013	7.85	7.97	36.0	980	1730	12	4	1530	86	2.1	1.0	0.04	4.48	0.37	515–835
BV-3	5/07/2013	8.15	8.05	37.8	1390	1720	7	3	1680	7	2.8	1.2	0.04	0.59	0.17	172–480
BV-3*	11/28/2013	8.28	8.18	36.1	1402	1650	7	3	1670	6	2.6	1.0	0.02	0.26	0.05	172–480
BV-9	5/07/2013	8.28	5.52	36.4	1512	1020	5	2	1140	5	2.6	1.2	0.04	5.12	<0.05	189–527
BV-9*	11/28/2013	8.36	5.72	35.0	1500	1040	5	2	1270	5	2.4	0.8	0.03	2.26	<0.05	189–527
BS-19	5/06/2013	8.36	3.46	35.3	1280	510	2	1	762	3	4.9	0.8	0.03	1.64	<0.05	362–566
BS-19*	11/27/2013	8.43	3.65	34.4	1317	540	2	<1	825	3	4.5	0.5	0.03	1.86	0.44	362–566
BS-36	5/06/2013	8.39	5.11	36.1	1963	745	3	1	1140	4	4.2	1.6	0.08	1.54	0.07	309–617
BS-36*	11/27/2013	8.49	5.30	36.5	1951	770	3	<1	1210	4	3.9	0.8	0.03	4.79	0.63	301–672
CD-8*	5/08/2013	8.49	4.61	30.2	2353	330	2	1	1000	4	4.9	0.4	0.05	2.28	0.41	460–804
CX-10*	11/25/2013	8.67	3.46	31.4	1256	539	3	<1	782	15	0.6	0.6	0.03	3.35	<0.05	200–442
PK-28*	11/25/2013	8.00	5.58	44.5	947	1260	9	1	1200	6	3.7	1.0	0.02	1.02	0.67	603–820
WP-3	3/24/2011	7.6	13.41	32.3	861	4280	38	9	2740	67	–	–	–	–	–	704–978
WP-3*	11/25/2013	7.90	7.00	42.9	939	1540	15	3	1400	31	3.9	1.4	0.02	36.0	0.06	704–978

Reproduced with permission from Baublys et al. (2015). All concentrations are listed in mg/L. An asterisk indicates that the measurements were taken at the same time as samples for microbial analysis.

**Table 5:** **Isotopic analysis of CBM production waters**.

	**Water stabel isotopes**		**Gas stable isotopes**					**Water age and tracer data**		
**Site ID**	**δ^18^O-H_2_O**	**δ^2^H-H_2_O**	**iδ^13^C-CH_4_**	**δ^13^C-CO_2_**	**δ^2^H-CH_4_**	**Δ^13^C_CO2-CH4_**	**Δ^2^H_H20-CH4_**	**δ^13^C_DIC_**	**^13^C age**	**pMC**
AG-13	–	–	−52.9	–	−233	–	–	–	–	–
AG-13*	−8.0	−49	−53.8	10.4	−216	64.2	167	13.4	>50,080	0.196
AG-31	−8.0	−50	−52.9	–	−226	–	176	14	50,700	0.182
AG-31*	−8.2	−51	−52.8	9.0	−212	61.8	161	11.7	52,200	0.151
BB-3*	−7.9	−48	−50.1	–	−216	–	168	16.8	45,860	0.331
BV-3	−8.4	−51	−51.3	–	−230	–	179	14.7	43,200	0.462
BV-3*	−8.6	−54	−51.7	–	−223	–	169	15.7	>49,720	0.205
BV-9	−8.8	−54	−50	–	−231	–	177	23.4	47,900	0.257
BV-9*	−9.0	−56	−50.4	10.8	−220	61.2	164	19.7	54,201	0.117
BS-19	−7.4	−46	−56.6	–	−234	–	188	18.2	43,650	0.437
BS-19*	−7.6	−50	−57	–	−223	–	173	13.3	53,250	0.132
BS-36	−8.3	−51	−53.6	–	−226	–	175	18.8	51,550	0.163
BS-36*	−8.4	−53	−53.8	9.6	−215	63.4	162	13.9	53,840	0.123
CD-8*	−7.4	−47	−49.9	–	−224	–	177	15.1	45,900	0.330
CX-10*	−7.2	−43	−44.5	11.0	−217	55.5	174	25.9	52,490	0.145
PK-28*	−7.4	−45	−49.5	6.2	−209	55.7	164	14.6	36,800	1.020
WP-3	−7.8	−47	–	–	–	–	–	–	–	–
WP-3*	−7.6	−46	−49.4	10.0	−216	59.4	170	14.2	47,680	0.264

Reproduced with permission from Baublys et al. (2015). An asterisk indicates that the measurements were taken at the same time as samples for microbial analysis.

## Discussion

Stimulation of additional biogenic methane from CBM production wells is likely to require a detailed understanding of the *in situ* microbial communities. Although a number of studies have characterized the microbial communities present in unperturbed CBM production wells, this is the first study to examine a CBM microbial community after hydraulic fracture stimulation. Clear differences in community composition were identified between PK-28 and wells that had not been exposed to hydraulic fracture additives. Metagenomic analysis revealed strong links between potential carbon substrates introduced in the hydraulic fracturing fluid and the metabolism of the dominant bacterial populations. These findings suggest that hydraulic fracturing has a marked effect on the composition and metabolism of CBM microbial communities.

The most significant compositional difference between PK-28, the hydraulically fractured well, and the 10 other CBM production wells was the presence of representatives from the bacterial candidate phylum *Aminicenantes* (11%) and class *Phycisphaerae* (9%) within the phylum *Planctomycetes*. These were present at <0.5% relative abundance in all non-fractured wells. Metabolic reconstruction of the *Aminicenantes*-PK28 and *Phycisphaerae*-PK28 genomes revealed the presence of genes for the degradation of galactomannon (i.e., guar), a common additive in hydraulic fracture fluid. Orem et al. (2014) have shown that the organic constituents of hydraulic fracture fluid can persist in the coal bed for several months after the fluids have been removed. Therefore, as water had only been extracted from the well for ~4 months, after having been allowed to incubate for 2 years, it is likely that galactomannan polymer still resided in the well (Struchtemeyer and Elshahed, 2012). In addition, estimates for the doubling time of microbes present in the deep subsurface biosphere under energy-starved conditions range from a few years to several millennia (Hoehler and Jørgensen, 2013; Onstott et al., 2014), suggesting that the microbial community structure of the well is likely to remain largely static for years after the galactomannan is removed.

Genes for the endo-hydrolysis of galactomannon (i.e., endo-mannases) were identified in the *Phycisphaerae*-PK28 genome, indicating that it is primarily responsible for the depolymerization of guar into short oligosaccharides monomers. These genes were identified in only one other member of the PK-28 community, *Ignavibacteriae*-PK28, and it is unclear why this microorganism is not more abundant. However, we can speculate that *Ignavibacteriae*-PK28 was more susceptible to the biocide. The presence of β-galactosidases and β-mannosidases in the *Aminicenantes*-PK28 and *Phycisphaerae*-PK28 genomes suggest that both microorganisms are able to use mannose and galactose produced by galactomannan degradation. In *Aminicenantes*-PK28, a putative phosphotransferase system for the uptake of mannose was identified that could be used to absorb mannose into the cell. No such system was identified in *Phycisphaerae*-PK28. The application of a peroxidisulfate breaker to partially hydrolyse the gelling agent in the hydraulic fracture fluid prior to removal from the well was likely to release free mannose and galactose monomers for consumption, as well as cell sulfur for *Phycisphaerae*-PK28. The presence of genes encoding pyruvate-formate lyase in *Phycisphaerae*-PK28, and formate dehydrogenase in *Aminicenantes*-PK28, suggests that formate, hydrogen, and carbon dioxide are major end products of fermentation. This would provide an avenue for a syntrophic association with the dominant hydrogenotrophic methanogens in the PK-28 community belonging to the family *Methanobacteriaceae*. In support of this hypothesis, a previous 16S rRNA gene amplicon based analysis of the water column of Sakinaw Lake (Canada) showed a statistical correlation between the *Aminicenantes* and hydrogenotrophic members of the order *Methanomicrobiales* (Gies et al., 2014). The unique ability of *Aminicenantes*-PK28 and *Phycisphaerae*-PK28 to ferment galactomannon in syntrophic association with a hydrogenotrophic methanogen, may have provided a selective advantage allowing these rare microorganisms to become enriched. Further support for the role of *Aminicenantes* and *Phycisphaerae*-PK28 in *in-situ* galactomannan degradation could be generated through the establishment of enrichment cultures seeded with CBM formation waters growing on galactomannan as a carbon substrate, potentially containing Kathon as a selective agent.

The ability to utilize a diverse array of polysaccharides, including guar-like polysaccharides, has been identified as a defining feature of *Planctomycetes* which are found in a variety of environments, including fresh and marine waters, hot springs, soils, and hydrocarbon contaminated environments (Yakimov et al., 2006; Abed et al., 2010, 2011; Lage and Bondoso, 2011; Tekere et al., 2013). Metabolic analysis of *Phycisphaerae*-PK28 showed that in addition to galactomannon, it has the potential to hydrolyse the xylose polymer xylan, as well as pectin. The ability to utilize complex sugars has been demonstrated previously in members of the *Planctomycetes* present within macroalgae-associated biofilms, and more specifically within the *Phycisphaerae* (Lage and Bondoso, 2014). For example, *Algisphaera agarilytica*, isolated from the surface of macroalgae, was shown to use agar, a galactose polymer, as a carbon source (Yoon et al., 2014), and *Tepidisphaera mucosa*, isolated from a hot spring, was shown to utilize pectin, galactomannon (i.e., locus bean gum), xylose, and galactose, but not xylan (Kovaleva et al., 2014). The *Phycisphaerae*-PK28 genome is consistent with these previous observations for members of the *Phycisphaerae*.

In contrast, very little is known about the ecology of the *Aminicenantes*, as no cultured representatives exist for direct characterization and metabolic analysis of three publicly available genome sequences has been extremely limited (Rinke et al., 2013; Gies et al., 2014; Sharon et al., 2015). Efforts to isolate the *Aminicenantes* or genomically characterize representative taxa are hampered by their low abundance in most communities. Previous analysis of over 3100 16S rRNA gene amplicon datasets mined from NCBI's sequence read archive (SRA) showed that the *Aminicenantes* were present in a quarter of all datasets, but they did not exceed 1% relative abundance in >99% of the data sets examined (Farag et al., 2014). Although present at low relative abundance, the *Aminicenantes* were identified frequently in fresh water, marine, and hydrocarbon-impacted environments, leading researchers to speculate on their role in these environments (Farag et al., 2014). Limited metabolic reconstruction of an *Aminicenantes* genome recovered from an acetate contaminated aquifer (Rifle, Colorado, U.S.A) revealed that it contained several glycosyl hydrolases (Sharon et al., 2015), but no investigation of the function of those genes was conducted. It was concluded that the Rifle Creek *Aminicenantes* may degrade carbon through either fermentation or aerobic respiration based on the presence of genes involved in aerobic respiration (respiratory Complex I, II, and III). This microorganism is also proposed to participate in hydrogen metabolism and assimilatory sulfite reduction. Analysis of a separate *Aminicenantes* genome recovered from Sakinaw Lake (Canada) revealed a partial set of genes for the Wood-Ljungdahl pathway (Gies et al., 2014). The authors speculated that the Sakinaw Lake *Aminicenantes* is capable of using this pathway in reverse to consume acetate and generate CO_2_ in syntrophic association with a hydrogenotrophic methanogen. In contrast to these previous findings, the population genome of *Aminicenantes*-PK28 does not indicate that it has the ability to perform aerobic respiration or produce CO_2_ via the Wood-Ljungdahl pathway. Instead, *Aminicenantes*-PK28 appeared to be capable only of anaerobic carbohydrate and amino-acid fermentation, producing CO_2_ through the oxidation of formate. Interestingly, a broad range of peptidase families were identified in *Aminicenantes*-PK28, suggesting that amino acid fermentation may be a key feature of its metabolism. For example, peptidases from family M23 capable of lysing the cell walls of other bacteria were identified in *Aminicenantes*-PK28 and may indicate that this microorganism acts as a scavenger of dead cells in CBM formation waters.

In this study, we have shown compelling evidence that specific additives within the hydraulic fracture fluid are responsible for a major shift in community composition which favors the enrichment of microorganisms from the rare biosphere that are able to utilize galactomannan. The observed enrichment of novel representatives of the class *Phycisphaerae* and candidate phylum *Aminicenantes* may also be coupled to their ability to work in syntrophic association with hydrogenotrophic methanogens and to the introduction of specific biocides into the well. It is possible that *Aminicenantes* and *Phycisphaerae*-PK28 are resistant to the Kathon biocide used in PK-28. Their resistance may result from the lack of genes known to be targeted by this biocide. Although both microorganisms possess 2-oxoglutarate dehydrogenase, other pathways may be used to accommodate its inhibition. For example, aspartate transaminase could be used to generate oxaloacetate for use in the TCA cycle. In addition, the unique cell wall structure of members of the phylum *Planctomycetes* (Fuerst and Sagulenko, 2011; Devos, 2014) may confer resistance to membrane disruption by Kathon. However, it is also possible, and perhaps more likely, that neither microorganism is biocide resistant, and instead may have simply recolonized the seam after the Kathon had degraded or dispersed to a low concentration. In this case, microorganisms best able to efficiently utilize guar as a carbon substrate would recolonize more quickly.

In addition to PK-28, wells WP-3, and AG-13 also appeared to cluster away from the other wells (Figure 3), indicating that they harbor atypical microbial communities compared to the rest of the Surat Basin. Neither of these wells were subjected to hydrofracture stimulation and neither showed enrichment in either *Phycisphaerae* or *Aminicenantes* lineages. Instead, both wells showed enrichment in thermophilic members of the bacterial family *Thermodesulfovibrio* and archaeal genus of methanogens *Methanothermobacter*, as well as members of the class OPB41 from the *Actinobacteria*. The observed enrichment in *Thermodesulfovibrio* and *Methanothermobacter* in wells with considerably higher than average temperatures (>40°C) is consistent with the optimum growth range of these lineages (Henry et al., 1994; Wasserfallen et al., 2000). Therefore, it is likely that these wells are atypical because their temperature is conducive to the enrichment of thermophiles. Additionally, WP-3 displayed a number of geochemical parameters such as pH, conductivity, and total iron that could be responsible for the observed microbial community shift. Additional basin-wide surveys are needed to identify the geochemical factors that govern CBM microbial community structure.

This study provides a basis for understanding how specific additives commonly used in hydraulic fracture fluid may alter CBM microbial communities. However, it is important to note that the findings of this study are specific to the set of additives used and may not be applicable to all CBM wells. Further, only one hydrofractured well was available for sampling. Therefore, examination of additional hydraulically fractured CBM production wells will be necessary to confirm these findings and determine how the microbial community will be affected under different stimulation scenarios. A longitudinal study is also warranted to document the community composition before and for several months after hydraulic fracture stimulation to determine if the community is capable of returning to an unperturbed state.

## Author contributions

SR, PE, DP, SG, and GT all contributed to the study design and helped to draft the manuscript. PE participated in sample collection and contributed to the analysis of the data. DP contributed to the bioinformatic analysis of this work, particularly in the area of population genome binning. All authors approved the final manuscript.

### Conflict of interest statement

The authors declare that the research was conducted in the absence of any commercial or financial relationships that could be construed as a potential conflict of interest.
